# Undignified Maternity Care During Childbirth: An Ethnographic Study on Midwives’ Perspectives in a Community Healthcare Centre in South Africa

**DOI:** 10.1177/23333936241273096

**Published:** 2024-10-14

**Authors:** Thandazile Adjunia Mayisela, Esther Lydie Wanko Keutchafo, Olivia Baorapetse Baloyi

**Affiliations:** 1 KwaZulu-Natal College of Nursing, Pietermaritzburg, South Africa; 2 University of KwaZulu-Natal, Durban, South Africa

**Keywords:** undignified care, childbirth abuse, labor, midwives, ethnography, South Africa

## Abstract

Undignified care of women during labor has been associated with increased preventable maternal morbidity and mortality. The purpose of this study was to explore midwives’ perspectives on undignified care of women in a midwife’s obstetric unit within a rural community healthcare center in South Africa. Using ethnographic methods, seven midwives were recruited to participate in individual interviews and participant observations were conducted in the unit. Three main themes describing undignified care were identified based on an inductive analysis of observations and interview data. These included a lack of respect for women’s bodily autonomy during labor, structural challenges in the provision of quality maternity care, and the lack of confidential care for women in labor and delivery. The study findings show that obstetric violence remains a serious crisis in the well-being of women during childbirth. Policy development by stakeholders in maternity care, including operational healthcare practitioners, should prioritize training, monitoring, and peer support on dignified care and curbing disrespect and abuse of women during childbirth, which seemed to be deeply rooted in the routine unorthodox treatment of childbirth.

## Introduction and Background

Global evidence suggests that undignified maternity care, which is defined as “any form of inhumane treatment or uncaring behavior towards a woman during labor and delivery” (WHO, 2018), is a public health challenge (Maya et al., 2018; WHO, 2018). According to the literature, both high-income countries and low- and middle-income-countries are not immune to undignified maternity care (Perrotte et al., 2020). Studies conducted in high-income-countries such as Germany, the United Kingdom, the United States of America and the Netherlands reported the prevalence of undignified maternity care between 17% and 25% (Baranowska et al., 2019). Whereas, studies conducted in low- and middle-income-countries such as South Africa, Tanzania, Nigeria, Ethiopia, and Kenya reported the prevalence of undignified maternity care in the form of disrespect and abuse by skilled birth attendants during childbirth at between 19.5% to 98.0% (Malatji & Madiba, 2020; Wassihun et al., 2018; Wassihun & Zeleke, 2018). Undignified maternity care has the negative consequence of discouraging women from seeking timeous care by skilled birth attendants during labor and delivery in their current and subsequent childbirths (Belizán et al., 2020; Burrowes et al., 2017; Maya et al., 2018). In addition, undignified maternity care could have adverse effects on a woman’s desire to have more children, women opting for late antenatal care bookings, home deliveries, and seeking assistance from non-skilled personnel. These actions could predispose the woman to prolonged labor, increased risk of post-traumatic stress disorder and postpartum psychosis (Belizán et al., 2020; Burrowes et al., 2017; Maya et al., 2018; Mwasha et al., 2023). Similarly, it predisposes the unborn baby to fetal distress and its extrauterine complications. Such dire consequences of undignified care are despite the WHO’s advocacy for facility-based delivery with skilled birth attendants in an attempt to curb childbirth-related fatalities (WHO, 2018).

Within low- and middle-income-countries, South Africa included, healthcare providers’ unprofessional behavior and attitudes have had counter-productive effects on the WHO’s efforts to advocate for facility-based deliveries, resulting in persistently high preventable maternal and neonatal mortality and morbidity (Jiru & Sendo, 2021; Saffie et al., 2018). In South Africa, women’s rights to dignified, respectful care are often violated through midwife battering and purposeful neglect (Bante et al., 2020; Dzomeku et al., 2020; Malatji & Madiba, 2020). Such dehumanized maternity healthcare practices violate women’s and girls’ rights to human dignity and respect, further impacting negatively on their maternity health-seeking behavior (Pickles, 2015).

### Aim

The aim of this research was to explore and describe midwives’ perspectives of undignified maternity care during childbirth in a community healthcare center in South Africa.

## Methods

Underpinned by social constructivism, focused ethnographic methods were adopted for this study (Cruz & Higginbottom, 2013; Rashid et al., 2019; Trundle & Phillips, 2023). Focused ethnography was used because it enables the study of the culture of a work environment and was, therefore, aligned with our aim to understand the sub-cultural practices of a small group of midwives as they performed their daily practices and interacted with birthing women. Moreover, in accordance with focused ethnography, the researcher typically possesses extensive expertise regarding the field of study and general culture. As such, there is less need for prolonged fieldwork to understand and interpret norms and practices in the study setting (Trundle & Phillips, 2023).

### Study Setting

The study took place in a 24-hour midwives obstetric unit within a rural community healthcare center. The midwives obstetric unit was selected because it is a receiving site for normal to low-risk pregnant and laboring women from the seven primary healthcare facilities in and around the residential area. High-risk pregnant and laboring women with conditions such as hypertensive disorders, obstructed labor, etc. are referred to the regional hospital for management of high-risk conditions either during pregnancy or labor (KwaZulu-Natal Department of Health, 2015). The selected Midwives Obstetric Unit has 18 adult beds, that is, a four-bedded admission unit, 10 observation beds and a four-bedded delivery unit (KwaZulu-Natal Department of Health, 2015). There is a one-bed neonatal transitional unit to cater for unforeseen neonatal complications, for example, neonatal respiratory distress. Human resources at the operational level in the unit include 14 advanced midwives, 10 basic midwives, 10 enrolled nurses, four nursing auxiliaries, and eight general orderlies. Personnel in the unit were divided into four teams to address patient needs. Each midwife/nurse worked 2 nights and 2 days per week. The selected facility also served as a Clinical Education and Training Unit for student midwives from the KwaZulu-Natal College of Nursing and the Durban University of Technology (KwaZulu-Natal Department of Health, 2020).

### Recruitment of Participants

The participants were selected through purposive sampling, which is aligned with focused ethnographic qualitative inquiry (Chopra, 2020). All registered midwives (basic and advanced) working in the labor ward at the study site at the time of data collection, on either day or night duty and responsible for rendering midwifery care to laboring women, were invited to partake in the study voluntarily. Among the 24 eligible registered midwives working on the unit, seven midwives agreed to participate in the study. Not included in the study were 10 midwives who verbalized their unwillingness to be part of the study, two midwives who were on annual leave, and one midwife who was on maternity leave.

In order to gain access to the research setting, the primary researcher (TM) organized a series of meetings with the facility managers. After these meetings, it was recommended that the researcher meet with the operational manager of the Midwives Obstetric Unit to discuss the study’s objectives and goals. Following this, meetings were planned by the operational manager to introduce the researcher and her study to all registered midwives on both day and night duty, affording everyone an opportunity to learn about the study. In July 2022, the researcher visited the setting four times in person, not for data collection purposes but to meet with and get to know everybody on each team (two visits on day and two on night duty). The visits allowed the researcher to build a rapport with the midwives and clarify any of their queries and misconceptions before the data collection commenced. It was difficult to book prior appointments with the participants because labor wards are unpredictable. Therefore, on the day of data collection, the researcher approached midwives who seemed less busy or free at the time. Each midwife was given an information letter to read alone, with the researcher providing any verbal clarifications required to obtain voluntary consent from participants to be observed and interviewed.

### Data Collection

Data were collected through individual in-depth individual interviews and non-participant observations. Combining observation with interviewing clarified the meaning of the events from the participant’s perspective (Hammersley & Atkinson, 2007; Streubert Speziale & Carpenter, 2007).

#### Observations

Non-participant observations using an observation guide were conducted by the researcher (TM) at least twice a week between 19:00–02:00 (night shift) and 07:00–19:00 (day shift) between August and November 2022, for a total of 102 hr. Day and night observations ensured that all study participants had an equal opportunity to be observed. Midwives and laboring women gave their consent to be observed. Women in labor were provided with clear information about the research study and their rights, including the option to decline observations. No patient declined the right to be observed. The researcher observed all the participants (*n* = 7) in the labor ward as they interacted with laboring women. The observation included observing midwives’ attitudes towards women and other midwifery professionals, communication skills with women and family members where applicable, non-verbal communication cues, power dynamics between midwives and women, and any absence of interaction was observed for appropriateness. Observations also included natural conversations, listening, and asking questions when necessary to clarify observations. All observations were recorded as field notes and were completed in the absence of the midwives to avoid mistrust.

#### Individual In-Depth Unstructured Interviews

Face-to-face in-depth individual interviews were also utilized to collect data from the five (*n* = 5) registered midwives who voluntarily consented and availed themselves. The interviews were usually conducted a day or two after the observations between August and November 2022. An interview guide, which was informed by data from the observations and qualitative ethnographic experts, was used in the in-depth unstructured individual interviews. All in-depth individual interviews were conducted in the study setting, in English as the preferred common language of proficiency by the participants, at a convenient time, in a relaxed private environment with no interruptions, minimal noise, good lighting, and ventilation. Only the interviewer and interviewee were present during the interviews. The in-depth individual interviews assisted to clarify and confirm what was observed during participant observations. The researcher asked open-ended questions based on the interview guide, such as: *What are your daily activities as a midwife when managing women during labor? How do you, as a midwife, ensure dignified care when managing women during labor?* When further probing was required, any of the following probes were used: “be as specific as possible;” “explain a bit more” which encouraged the participants to elaborate further, thus enabling the researcher to elucidate current and relevant discussion points. However, in their (midwives) description of “dignified care,” “undignified care” was revealed. Interviewing techniques such as summarizing, encouraging, and paraphrasing were utilized, allowing for confirmation of data.

At the end of each interview, the researcher summarized the core points and issues that were discussed, and each participant was given an opportunity to add or change any statements until she was satisfied with the comments. Each interview lasted approximately 30 and 45 min and was audio-recorded and transcribed verbatim. Handwritten field notes and memorandums were kept to provide a system of backup information throughout the data collection (Pope, 2005). Pragmatics related to available time and resources limited data collection and time spent in the field as can be the case in ethnographic studies like this (Cruz & Higginbottom, 2013; Jones & Smith, 2017). Nevertheless, the data collected through observations and interviews enabled a detailed description of the culture of maternity care in the study setting.

## Ethical Considerations

Ethical permission to conduct the study was obtained from the University of KwaZulu-Natal’s Biomedical Research Ethics Administration (BREC/00002519/2021). Aligned with Section 18 of the Protection of Personal Information (POPI) Act, which alludes to “notification to the data subject when collecting personal information” (POPI Act, 2020), registered midwives were informed at the beginning of every shift whenever the researcher was present to conduct observations, and their rights to withdraw from the study at any given time were emphasized. Furthermore, the research rights of the registered midwives were respected and protected throughout the study. All registered midwives in the labor ward were provided with an information sheet explaining the research aim, methodology and their rights prior to providing written consent. Despite the researcher’s assurance that all data, including recorded data, would be kept confidential and not linked back to the participants in any way, two registered midwives declined to participate in the individual interviews. They did agree to be observed and were allowed to do so without consequences.

Most research has the potential for either physical or emotional risks (Resnik, 2015). The researcher anticipated that midwives could experience emotional distress as they revealed information they might consider sensitive and private, such as cases involving maternal deaths as a consequence of undignified care or their own participation in/or witnessing colleagues rendering undignified care. As a precautionary measure, arrangements for counselling, if required, were made with counsellors from the Employee Assistance Programme. Any participants who experienced any form of psychological distress could be referred to the Employee Assistance Programme within the facility for free psychological therapy. Fortunately, none of the participants verbalized nor displayed any psychological or emotional distress. Individual interviews were not linked to the midwives’ names. Participants were not addressed by their real names during the interviews (Resnik, 2015). Self-selected pseudonyms were used during individual interviews. Information from observations and individual interviews was not linked to the midwives’ names. During data collection, the researcher (TM) observed undignified care rendered by the midwives, which contravened the South African Nursing Council (SANC) Code of Ethics. In an attempt to address observed unethical conduct by the midwife participants, the researcher reminded them that undignified care is a serious offence, according to the SANC. As a supportive intervention, the researcher printed copies of the Scope of Practice of Midwives and the Acts and Omissions as stipulated by the SANC, in an attempt to offer reorientation to the midwives about their roles and responsibilities in rendering safe, dignified maternity care.

## Data Analysis

Data analysis was guided by the inductive approach suggested by Babchuk (2019). The 10-step approach shares commonalities with systematic processes of data analysis used in ethnographic studies such as identifying and classifying the data using descriptive labels or codes, exploring the data for patterns, progressing to explanations of the patterns, and memoing reflective remarks (Cruz & Higginbottom, 2013). Data management was achieved through the electronic software NVivo version 12. Transcripts were shared with the research supervisor as the co-coder and further discussed with the specific midwifery informants and co-authors to enrich the researcher’s understanding of the data to ensure consistency.

Furthermore, data from participant observations were initially arranged in hard copy tables with thick descriptive notes of interactions between midwives and women in the left-hand column and generated descriptive labels in the right-hand column. In Step 2, the researcher and the co-coder focused on re-familiarizing themselves with the data through a close reading of transcripts and listening to the recordings to strengthen their connection with the data. A record of personal reflective memos were also kept. In Step 3, the researcher and supervisor conducted a second read of transcripts and observational notes, independently beginning initial or open coding through word-by-word and line-by-line coding techniques. The midwives’ perspectives on undignified care of women, which occurred across interviews, field notes and observational notes, were noted and underlined, forming text segments. Probable headings were assigned that best described the text segment, forming codes. This process was repeated several times, allowing the researchers to get closer to the data while fine-tuning the codes. The researchers proceeded with steps 4 and 5, looking for similarities among the codes, narrowing or “winnowing” the codes to eliminate overlap and redundancy, and focusing specifically on codes that address the research aim. The related codes were grouped to identify sub-themes. In this way, a manageable number of themes and sub-themes were generated to describe the culture of maternity care.

## Trustworthiness

Academic rigor was ensured following Lincoln and Guba’s (1985) and Shenton’s (2004) strategies of credibility, dependability, confirmability, and transferability. Several measures were taken to ensure credibility. These included prolonged engagement by the researcher with the ethnographic hosts and cultural informants at the setting, validating collected data against results with the participants and matching it against verbatim transcripts. In addition, the themes and sub-themes were discussed among the researcher and her supervisor regularly to enhance peer scrutiny. This was in addition to confirming the codes and themes/sub-themes with the co-coder and critical reader.

In terms of the researchers’ positionality, the lead author (TM) was not an insider to the study setting. Although all of the researchers were outsiders (Sybing, 2022), and not known to the participants nor familiar with the study setting, the lead author (TM) had an extensive clinical background with more than 20 years’ experience as a clinical midwife. Therefore, she had an in-depth personal understanding of the contextual elements mentioned by the midwives, which continued to shape the research she had undertaken, informed qualitative interview guides, and interviewing techniques inclusive of data analysis which strengthened the credibility of this study.

To ensure dependability, the study commenced with a detailed and well-articulated proposal and included data quality checks with qualitative ethnography experts. Interviews were immediately transcribed verbatim to validate information identified through observations; and data collection, analysis and examination of observational notes were closely monitored through regular meetings with the research team. Confirmability was supported through an inquiry audit, detailed field notes, data and methodological triangulation, authenticating transcribed data with the participants and involving an experienced independent co-coder who also supported the development of themes and sub-themes. Lastly, transferability was ensured by providing participants’ demographics, a thick description of the phenomenon under study, study methodology, context, settings, procedures, and findings as recommended by Shenton (2004). Furthermore, the study provided a detailed description of the results to enable comparison to similar contexts and settings. There are four types of triangulation from which an ethnographer can choose: data triangulation, investigator triangulation, theory triangulation, and method triangulation (Streubert Speziale & Carpenter, 2007, p. 381). This study used data triangulation (data collected at different times, from different people in a setting) and methodological triangulation (participant observation, unstructured interviews and field notes to determine what informants said was what they did during their daily work).

## Findings

### Profile of the Participants

Seven midwives were observed, of which five were interviewed. All the participants were female; six were advanced midwives, and one was a general midwife. Their years of experience ranged from 8 to 40 years.

### Research Findings According to Themes and Sub-Themes

Table 1 represents a summary of developed themes and sub-themes.

**Table 1.** Summary of Developed Themes and Sub-Themes.

Themes	Sub-themes
1. Lack of bodily autonomy	1.1 Non-participation in decision-making during labor and delivery
	1.2 Unconsented clinical interventions throughout labor and delivery
2. Structural challenges in the provision of quality maternity healthcare	2.1 Non-confidential care of women in labor and delivery
	2.2 Insufficient space for women’s preferred support person (birth companion)
3. Obstetric violence	3.1 Physical abuse of women during labor
	3.2 Verbal and emotional abuse, condescending and infantilizing of women
	3.3 Substandard pain management during labor

#### Theme 1: Lack of Women’s Bodily Autonomy

All participants referred to women’s “lack of bodily autonomy” during labor as the main challenge in the provision of dignified care. They emphasized the significance of their role in decision-making, guiding and leading the childbirth process while “forcing” women to follow instructions. The participants noted that it was essential to control the birthing processes to prevent complications and possible adverse outcomes. The researcher observed that there was evidence of domination in how each midwife related and interacted with women in the labor room. All participants adopted a culture of minimal information sharing in their interactions with women. Minimal information sharing kept women powerless and unable to participate in their birth processes. The first sub-theme that developed was a culture of women’s non-participation in decision-making.

##### Sub-Theme 1.1: Non-Participation in Decision-Making During Labor and Delivery

This sub-theme developed early from responses made by informants and observed interactions between women and midwives. Interviewed midwives said they did not allow women to participate in decision-making during labor and delivery as it was not the norm. Midwives mentioned that women needed to be “told” what to do, and that was evident in their roles, beliefs and values when rendering what was believed by them (midwives) to be professional skills and dignified care. The following quotes highlight perceptions of midwives:

I never allow them (women) to make any decisions during labour or delivery I tell her [the woman] that I’m going to assess her abdomen, I’m going to do the PV (per vagina) examination, then I tell her; yes, you are in labour, you are 3 cm now, and most women do as they are told, but we tell them in a polite manner. [Midwife 1, F, 14 years of experience]

There is routine care that works for both young and old women during labour, so we do not ask for their opinion, we decide for them and those who have different requests are advised to join everyone because routine methods are reliable. [Midwife 4, F, 8 years of experience]

As suggested by most informants, women took instructions from midwives and never raised any objections or complaints. One midwife emphasized the importance of keeping to the same well-known and trusted ways of caring for women during labor. Therefore, women with diverse needs and challenges would be advised to accept routine treatment methods at the facility. During observations, participants were observed leading the interactions with women, and women would follow instructions throughout the labor process.

Participants in this study further explained that in the labor room, there were two lives (that of the mother and the unborn baby) to take care of, and taking requests made by women or allowing women to take part in decision-making could lead to potentially negative outcomes. The following extract from an interview illustrates these beliefs:

Some women do ask for injection for pain when they get very painful contractions, and we remind them to take deep breaths. We do not use any strong injections because labour is natural, and those injections can be dangerous to the new-born. . . [Midwife 4, F, 8 years of experience]

Participants indicated that some women do attempt to involve themselves in decision-making during labor and delivery by suggesting what they want and prefer, for example, asking for pain medication, if they can rest in bed when they are feeling tired, or asking to try any other delivery positions than the traditional dorsal position. The extracts below indicate how midwives opted not to involve women in any decision-making during labor despite their attempts:

All women are made to deliver in the dorsal position, and if they want to squat we explain to them, (we do) not allow it and they agree with us. [Midwife 3, F, 40 years of experience]

I have had women, especially those experienced multiparous ones, wanting to deliver babies while they kneel. The thing is, I cannot see the baby and that can lead to baby rushing out and getting injured. . . We put all women on their backs for delivery to avoid complications. [Midwife 4, F, 8 years of experience]

Minimal information sharing and the culture of non-participation by women presented the sub-theme of lack of consent during labor, and the next sub-theme developed.

##### Sub-Theme 1.2: Unconsented Clinical Interventions Throughout Labor and Delivery

During participant observation, the researcher observed that in most instances, midwives rendered care without requesting women’s consent. Most midwives confirmed this observation during interviews, stating that whenever they had to perform episiotomies, they would cut the perineum during the height of a uterine contraction without asking for consent, citing a lack of time to ask a woman for any form of consent. The midwives emphasized that they did not consider cutting episiotomies to warrant any form of consent, and indicated this was part of their instruction in midwifery colleges. The following are some of the extracts from the interviews:

I only perform an episiotomy when there is a rigid perineum or when there is a difficult delivery, not just routinely and I do not get time to ask the patient for consent, it is sort of an emergency, it’s not planned. . . Patients know about such things, they know that it is done to protect the baby from complications so why ask for consent. [Midwife 1, F, 14 years of experience]

. . .If I must cut an episiotomy I do it, if I must put up a drip I do it I don’t ask the women, but I do tell them first. . .this is part of routine care it does not need consent. [Midwife 1, F, 14 years of experience]

Participants further discussed interventions such as manual evacuation of the uterus, which was performed routinely without any pain medication and for no clinical indications. Usually, evacuation of the uterus is indicated when products of conception have been retained, such as in the case of a placenta accreta. The following extracts came from participants’ interviews:

I do manual removal of clots following delivery of the placenta, removal of clots prevents postpartum haemorrhage which may be caused by big clots, okay uhm, no pain medication is given. The patient does not know that it’s done but they do scream for a short period of time. . . [Midwife 3, F, 40 years of experience2]

. . . cleaning the uterus by scooping clots out prevents retained placental membranes, unfortunately we can’t give her (the woman) any injections because she must start breastfeeding. It’s done fast, it’s common practice. . . yes. [Midwife 2, F, 9 years of experience]

One of the participants further added:

Birth (Human immunodeficiency Virus [HIV]) PCR (Polymerase Chain Reaction) test is a common procedure, done almost every other day, and mothers talk about these things, they know it. When we bring the collecting kit, mothers know already. . . there isn’t more explanation needed, you know. We keep it hush-hush to protect them (women) after all, you know. [Midwife 5, F, 21 years of experience]

Participants in the study concurred that spatial arrangements in the labor room presented challenges regarding privacy and confidentiality issues related to both the woman’s physical care and as well as protecting her personal information. In this context, women’s dignity could be compromised if sensitive personal information was revealed to or overheard by others.

#### Theme 2: Structural Challenges in the Provision of Quality Maternity Healthcare

Participants in the study concurred that spatial arrangements in the labor room presented challenges regarding privacy and confidentiality issues for both the woman’s physique and personal information. Women’s dignity of care could be violated should sensitive personal information be shared unscrupulously. The first sub-theme deals with confidentiality and privacy challenges identified by participants. The labor ward where the women were kept during the first stage of labor was an open plan, and the midwives shared the same space with them.

##### Sub-Theme 2.1: Non-Confidential Care of Women in Labor and Delivery

Confidentiality of care in healthcare refers to rules that limit access to private information about a patient, both verbal and written information. The chosen facility’s physical layout was such that midwives and patients were always in the same open space at any given time, and any conversations, either among personnel or between patients and personnel, would always be within earshot of everybody. Patient files were kept away from patients to ensure confidentiality. This was done to prevent unnecessary exposure of personal information to other patients, relatives and healthcare practitioners who were not directly involved with the labor ward. Except for patients’ files, the ward’s arrangement was concerning for some midwives, as reported below:

Women in labour and those who are postpartum share the same space, this is a small clinic, and it is difficult for them not to know what is going on with the next person. It is a real problem, but we try to maintain privacy, we do the best we can you know but it is not easy. [Midwife 4, F, 8 years of experience]

We use screens to protect patients but if we say something there is no soundproof, everyone who is in the room can hear what is being said. It’s just like that. . . (shrugs shoulders). [Midwife 3, F, 40 years of experience]

Well. . . (paused), this is a huge room as you can see, patients get to hear some information about each other sometimes. We try not to mention anything serious in the ward, we have to be careful of what we say and do at all times. . . this is not a big hospital like. . .but sometimes it’s hard if like you want the mother to see or hear that you giving her baby nevirapine syrup. . .other mommies can see you and make conclusions about the HIV status. [Midwife 1, F, 14 years of experience]

The Midwives Obstetric Unit had a nurses’ table near the entrance that served as a nurses’ station, and women in labor occupied couches at the opposite end of the ward. Conversations between the midwives and the women would take place over a distance unless the nurses walked across to the vicinity of the couches. There were 12 beds arranged in two rows, six on each side of the unit, and each had curtained screens with rails anchored on the ceiling. The table could accommodate two people at the most, and it was near the entrance with one armchair and a plastic chair. There was minimal space around the table. No place was identified as an office for the unit’s operational manager or administrative area.

During participant observation on this particular day, two mothers had given birth, and their babies needed birth HIV-PCR tests due to exposure to HIV. Blood samples from the babies were obtained in an open space, and the researcher was allowed to witness the procedure performed on both babies. Some participants showed concern, as witnessed in the extracts above (sub-theme 2.1).

##### Sub-Theme 2.2: Insufficient Space for Women’s Preferred Support Person

Participants expressed concerns about the size of the labor ward and the inability to accommodate women’s chosen companions. Participants were aware of the benefits of having a companion for the woman and her unborn child in improving birth outcomes and positive birth experiences. The researcher probed participants on their perceptions of the provision of support to women in the facility, and the following excerpts and extracts were identified from observational and interview transcripts, respectively:

Some patients ask if their mothers or grandmothers can stay with them during labour, they can’t because there is no space, the space is limited, as you can see there is no privacy for two people to be together. . .the infrastructure does not allow for this. . . . but over and above we don’t want supporting people, they are not here to support their loved ones but rather to observe and challenge what we are doing, I’m the trained one I’m in charge during labour. [Midwife 2, F, 9 years of experience]

Relatives can stay for about ten minutes only, but they are not allowed to sit in, so there is no continuous support unfortunately because of lack of space and overcrowding, and we are always short-staffed, it works well for us though that they are not allowed to sit in, they can be irritating sometimes. [Midwife 3, F, 40 years of experience]

It is a bit uncomfortable to be working and there is someone watching you like police or a spy. . .. I personally don’t like it. . .These relatives record what we doing the next thing it all over social media. . .**‘phansi’ (no way)** with a birth companion. [Midwife 1, F, 14 years of experience]

According to the participants, some women would request that their family members stay with them during labor, and women would be told that the labor ward provides only for the mother-baby pair and relatives or friends cannot be accommodated.

#### Theme 3. Obstetric Abuse

Obstetric abuse was evident in both interviews and observations and was reflected in both midwives’ actions and communications with laboring women. The abuse involved intentional physical actions, insulting verbal interactions and sub-standard pain management. Each of these types of abuse were used to control women during labor and are described in the following sub-themes. The first sub-theme under obstetric abuse to develop from the participants’ interviews and interactions with women involved intentional bodily injury by the midwives towards patients as a control measure.

##### Sub-Theme 3.1: Physical Abuse of Women During Labor

Participants acknowledged that there was a symbiotic relationship between poor pain management and the incidence of physical abuse in their interviews:

Some women do not push the baby out, and it gets stuck in the outlet. Fundal pressure is used in such cases, not every day though. . .with those few cases, yes. That is when sometimes we push the woman’s thighs apart, and we slap them but not across the face. Women cooperate when they are frightened, otherwise babies will die every day [Midwife 1, F, 14 years of experience]

Before coming to this clinic, I used to give local pain injections then cut, but now I wait for her to ‘push’ and then I cut quickly without analgesia, it’s fast. All women scream out loud when being cut but it is over very fast. I hit patients during pushing I don’t wanna (want to) lie to you, just slight slapping on the body not the face. This is a lot of work. . . [Midwife 3, F, 40 years of experience]

All participants agreed that hitting patients worked to their advantage, although they were not in favor of it. They believed their options were limited. They emphasized that slapping was usually limited to the thighs and avoided hitting the face. Light slapping was not harmful, and the aim was to encourage women to do the right thing, according to the participants. Participants strongly believed that giving local analgesics before performing an episiotomy was not a clever idea, and giving a woman an opportunity to consent was not clinically ideal. The belief was that women could not differentiate between pain caused by stretching the perineum due to extension of the fetal head and that caused by surgically cutting the perineum.

Participants perceived the use of fundal pressure, forcing women’s legs apart, and labial pinching with forceps as important actions to prevent new-born deaths.

The second sub-theme under obstetric abuse developed from the participants’ interviews and interactions with women encompassed the use of harsh words and psychological mistreatment of women during childbirth

##### Sub-Theme 3.2: Verbal, Emotional Abuse, Condescending, and Infantilizing of Women During Labor

Most participants mentioned that they become verbally abusive to laboring women to gain their full cooperation. Condescending and infantilizing were reported through all four stages of labor, and the participants considered their actions to be polite. The following extracts are supportive of this statement:

We try to maintain dignity but in the second stage of labour sometimes we ‘fight’. . .we sometimes like most often become verbally abusive and use strong language to the women. Yes, those who are not cooperating, it’s then that we raise our voices and give them a hostile look to control the situation and help them. [Midwife 1, F, 14 years of experience]

Yes, sometimes we do yell at patients, especially (the) ‘small young ones’ who are pregnant and not married. . . they do not listen. I tell them that we are not allowed to hit them therefore, they have to do as we tell them. We tell them to take a deep breath and place the chin on the chest while looking at the vulva where baby is coming. [Midwife 2, F, 9 years of experience]

During labour admission and delivery, some women will be asked by midwives why they keep on having more children, and if they are doing it for SASSA (South African Social Security Agency) Child Support Grant purposes. [Observation: December 6, 2022]

Most of the women who came to seek healthcare services at the facility were youthful, while the midwives were of mature age (≥35 years old). These dynamics played out in interactions between personnel and patients. Experienced midwives suggested that young mothers were “just girls.” Patients would be addressed as “Little one” or “The little one who does not listen” in an autocratic tone. One young mother was addressed in an infant-like voice, pronunciation, enunciation and intonations, and the young mother was observed to be smiling but fearful throughout the interaction. Although the young mother was infantilized, it seemed like she had accepted the situation even though she appeared uncomfortable.

The third sub-theme under obstetric abuse developed from the participants’ interviews and interactions with women involved intentional deprivation of pain management by the midwives towards laboring women.

##### Sub-Theme 3.3: Substandard Pain Management During Labor

Participants’ views and perspectives on pain management during labor were suggestive of sub-standard care, disregard of guidelines and resource constraints, as evident in the following extracts:

We observe them (women), we don’t give them anything for pain until they give birth, then they can have some paracetamol tablets. They do complain a lot (about pain) especially when the (foetal) head is delivered. [Midwife 2, F, 9 years of experience]

Some women do ask for injection for pain when they get very painful contractions, and we ask them who told them we give injections here. . .I mean sometimes is simply because we don’t have that pethidine.. . .I don’t have time to explain. . .and if I were to give it (pethidine) and it affects the foetus where will I get resources to resuscitate the baby. . .its better I refuse to give it and let the woman suffer with pain to save my own job. [Midwife 4, F, 8 years of experience]

It was evident that all participants shared the same views and beliefs when it came to the use of pharmacological pain management. They relied on deep breathing and relaxation exercises as seen below:

Deep breathing during contractions helped in most cases but some patients get out of control towards the end of labour. We are forced to shout and scold them, not too much. . . but we do not hit them because it is not allowed. Some patients need to be held down when they push the baby out, we do it for their safety. [Midwife 2, F, 9 years of experience]

Relaxation and breathing exercises work during early stages of labour, there is nothing more that can be done in the late stages though, I know we are not doing enough but we worry about complications caused by strong drugs, new-borns can die you know. [Midwife 3, F, 40 years of experience]

Participants suggested that women were strong by nature, and it was expected of them to cope with labor pains. Some expressed fear of side effects. Participant observation produced the following excerpts:

To create a positive experience and improve the quality of maternity care pain management should be effective. Watching women cry and scream due to labour pains makes me feel extremely uncomfortable but I need to keep to my researcher role. [Observation and memo: September 3, 2022]

Two primigravidas were in labour, one kept looking in the direction of the midwives’ table each time she experienced uterine contractions, and her eyes were wide open as if she was anxious. She stated that pain was too much for her, and nothing was done for four and a half hours when I was in labour ward. [Observation: September 4, 2022]

Participants indicated that they were aware of substandard pain management during the first and second stages of labor at the facility, but they maintained refusal was done with good intentions for the safety of the baby. Particularly, the participants were concerned about a flat baby as a consequence of opioid (pethidine) use. This is despite the availability of resuscitation equipment at the setting in the event of a flat baby due to the side effects of opioids. All the participants spoke against the use of injectable opioids in the labor rooms, and there were no explanations provided for withholding inhaled analgesia such as Entonox (nitrous oxide).

I know and I’m aware that I’m supposed to give pain medications, but I don’t. . .see the issue with pethidine for example is that it affects the unborn baby too, you know it causes respiratory distress. . .like really why should I stress myself with a flat baby after birth [Midwife 2, F, 9 years of experience]

Yes, we do have resuscitation equipment and capacity to resuscitate but it is unnecessary stress just in the name of pethidine, labour pains are natural and waiting for that ambulance to transfer out is tedious, one can wait for six to eight hours risking losing the baby over pethidine no, it is not worth it. [Midwife 4, F, 8 years of experience]

Refusal to provide pain medication to those women who needed it led to poor cooperation by women during the second stage of labor. It forced participants to shout at women and slap their thighs while forcing them apart. The midwives stated that yelling and holding the thighs down was better than treating an asphyxiated baby and dealing with possible neonatal death.

I better yell and shout than to resuscitate [Midwife 1, F, 14 years of experience]

## Discussion

Although dignified care of women during labor has been identified as a priority, the findings of this study indicate that many women in the study setting were not receiving this care. Instead, a culture of undignified care in the midwifery unit was predominate. This culture was described under three themes: (1) midwives’ normalized practices that violated women’s right to bodily autonomy, (2) the physical layout of the labor room unit that made it impossible to offer confidential care or accommodate a birth companion for laboring women, and (3) the use of abusive practices by midwives as control measures during labor.

In the first theme, normalizing the violation of women’s right to bodily autonomy in the form of non-participation in decision-making about their care was a daily occurrence in the study setting. Participant midwives believed that they were in charge of the women’s labor and delivery processes and that they (women) needed to be “told what to do and not to do” hence not allowed to make any autonomous decisions about their maternity care. The study further evidenced that laboring women in the study setting were younger and mostly teenagers, which prompted midwives to “auto-tune to parenthood” (to assume parenting roles), further adding to women’s non-participation in decision-making about their care. This particular study finding is congruent with Sadler et al. (2016), who attested to midwives often assuming parenting roles towards teenagers and younger laboring women, which made it harder, if not impossible, for the women to voice out their preferences during labor and delivery, resulting in their non-participation in decision making.

In addition to non-participation in decision-making during labor and delivery, women in the study setting were subjected to non-consented clinical interventions at the hands of the treating midwives. Midwife participants knew and agreed that failing to inform women about medical procedures done on them violated their basic human rights, such as the principles of justice and non-maleficence. However, midwives were not deterred despite their awareness that they were in violation of women’s rights, as well as their own scope of practice, which advocates for patients’ informed consent regarding medical procedures, or anything related to their care (SANC, 1984). Midwives’ consciousness of their own acts and omissions did not stop them from performing non-consented invasive and non-invasive procedures on women. Consistent with Khumalo and Rwakaikara (2020), midwives in the study setting believed that performing non-consented clinical interventions kept things under control. Of interest in this study is that the performance of non-consented clinical interventions was identified as a favor by the midwives to ensure satisfactory delivery outcomes. According to the midwives, cutting an episiotomy when the delivery is being obstructed by the tightness of the perineum is not only mandatory but controls the delivery to ensure a healthy baby. Hence, they see no need to ask for consent from the women. The participants believed that by so doing, the women would go home happy with a live baby. Ultimately, they (midwives) will protect their own jobs and professions through avoidance of litigation as a consequence of patient safety challenges.

The second theme focused on the role of infrastructure necessary for midwives to render quality maternity care, a problem observed especially in poorly resourced countries. In these settings, overcrowding, inadequate clinic facilities, lack of equipment and shortage of personnel are commonly identified as partially responsible for inhumane care of women during labor and delivery, which serves to exonerate the midwives and other healthcare providers to a certain degree since the circumstances are beyond their control (Boakye et al., 2021; Mwasha et al., 2023; Yakong et al., 2010). Unfavorable physical layouts of labor rooms that compromise women’s privacy also inhibit the midwife’s ability to render safe and quality midwifery care, a finding supported by other studies conducted in resource-constrained settings (Boakye, 2022; Boakye et al., 2021). The labor room layout in this study setting was suboptimal for providing high-quality maternity care according to the South African Constitution, which strongly advocates for the protection of its citizens against violation of their rights to privacy and personal dignity (South African Government, 1996). Confidentiality in healthcare is a legal and ethical obligation to protect health service users’ personal and health-related information (South African Government, 1996). Despite this, the midwives in the study setting were required to perform their daily duties in a shared open space, which compromised women’s privacy during physical examinations and confidentiality of their personal and health-related information placing midwives at risk of disciplinary action by the SANC (2014).

Non-confidential care coupled with insufficient space to accommodate birth companions was identified as a fertile ground for undignified and disrespectful maternity care in the study setting. Midwives were aware of the benefits of having a birth companion for women. Evidence in the literature indicates that the presence of a birth companion lowers fear, anxiety, pain, and agitation in the women, making them amenable to direction while simultaneously improving midwives’ attitudes towards the women, which, ultimately, could improve the overall outcomes of childbirth (Brenes Monge et al., 2020). Midwives at the setting unreservedly discouraged the presence of the birth companion, meaning no one was allowed to have a birth companion. Besides the infrastructural challenges, the main reason cited by the midwives was their negative attitude towards the presence of outsiders in the labor ward, perceived as being there to “spy” on staff rather than as a birth companion. This particular study finding concurs with Dzomeku et al. (2020) and Sadler et al. (2016), who discovered that the midwives believed that the presence of a birth companion held a potential to witness the mistreatment of women by the midwives. It was astounding to listen to the midwives alluding that, in the presence of a birth companion, they would be unable to use physical force to fast-track labor and delivery.

A further observation at the study site was that banishing birth companions made it easy for midwives to physically abuse women without fear of anyone witnessing the offence. In addition to what the researcher observed, the midwives at the study site admitted to physically abusing women during labor and delivery. According to them, physical abuse was perceived as a much-needed and necessary strategy. The rationale behind it was to enhance labor and delivery, similarly assisting women in giving birth to healthy babies. Local and international authors have also reported physical abuse to laboring women ranging from forcefully holding women’s thighs apart, forcing women to feel the baby’s head following crowning, cutting the perineum without local analgesia, tying legs on lithotomy poles for prolonged periods, to slapping the thighs (but not the face), and pinching the thighs and labia with artery forceps (Bohren et al., 2019; Dzomeku et al., 2020). Midwives in the setting knew that these actions were wrong, cruel and abusive. However, in their own words, they had no intentions of stopping such acts anytime soon. According to them, they did not see a need to stop actions because they resulted in a satisfactory outcome of a “healthy baby” and “healthy mother.” This is congruent with Mehretie Adinew et al. (2021), who reported that some participants considered disrespect and abuse acceptable as they prioritize medically indicated procedures over patient-centered care. For these participants birthing a live baby to a healthy mother is a top priority to avoid negative outcomes. The aforementioned actions by the midwives were not part of either routine or evidence-based midwifery practice. They are classified by Bohren et al. (2019) as inhumane, cunning and showing a lack of conscience from the midwives’ side.

In theme three, it is evident that physical abuse has been normalized by the participants at the study site as the most generic form of undignified and disrespectful treatment. Aligned with Khumalo and Rwakaikara (2020), some women in the study setting could be heard praising and thanking the midwives for rough handling them when the baby was born, which was an indication that patients had accepted and normalized physical abuse. Similarly, the midwives responded by accepting praises and agreeing with the women that life could have been lost if it had not been for her (midwife). Undignified and disrespectful care in the form of physical abuse in the setting was further exacerbated by the lack of systems in place to monitor incidents of unlawful handling of women during labor.

Physical abuse did not happen in isolation; it occurred simultaneously with verbal abuse. Effective and therapeutic communication is one of the key components of *Respectful Maternity Care* (Bante et al., 2020). However, in the study setting, midwives possessed poor communication skills, evidenced by how women were often humiliated and called names by the treating midwives, especially during the second stage of labor. Cruel and shameful comments were made by the treating midwife about women’s parity and marital status. Primigravidas were blamed for premarital childbearing, while multigravidas were ridiculed for bearing more children in an attempt to access the Child Support Grant from the South Africa Social Security Agency (SASSA) (2014). It was interesting for the researcher to listen to the midwives shifting the blame for verbal abuse to their own colleagues. Each midwife saw the next midwife as the worst when it came to verbally and emotionally abusing laboring and delivering women at the study site. However, midwives continued with the verbal abuse of the women, despite evidence from literature emphasizing that these personal attacks and heartless judgmental comments may have undesirable consequences, such as discouraging women from going to the healthcare facilities for childbirth, leading to a delay in seeking skilled birth attendant’s expertise, and disruption in continuity of care when women hop from one facility to the next hoping for better services (Downe et al., 2018). The above-mentioned ill-fated and potentially dangerous situations could indirectly contribute to preventable maternal mortality, morbidity, and near-misses (permanent disabilities) due to negative outcomes resulting from unsupervised home births (Kwame & Petrucka, 2020; Tenaw et al., 2022).

Laboring women at the study site were not only physically and verbally abused. They were also refused any medicinal analgesia due to midwives’ beliefs that labor pains were “natural” and that recommended opioids caused more harm than good, particularly to the unborn baby. Despite the availability of resuscitation equipment, the participants cited personal preferences, such as averting the stress of neonatal resuscitation or upward referrals in cases of severe neonatal birth asphyxia. This study finding is congruent with Khumalo and Rwakaikara (2020) and Dzomeku et al. (2020), where midwives refused to administer opioids or administered low doses of opioids because of their own anxiety about side effects, particularly to the unborn baby and neonate. Refusal to administer opioids was despite evidence in literature emphasizing that opioids increase the pain threshold and improve the health outcomes for women, therefore decreasing the incidence of maternal morbidity and mortality, including near misses (Olza et al., 2020). Conversely, undignified maternity care coupled with withholding opioids causes fear and anxiety, which may prolong labor and delivery (Olza et al., 2020).

### Strengths and Limitations

The study findings indicate that obstetric violence remains a serious threat to the well-being and survival of women during childbirth. Although data collection was limited to one facility, detailed observations and interviews provided a rich description of the care laboring women received. Nevertheless, the findings are not generalizable beyond settings that are similar to the study context.

## Recommendations

The study findings suggest a number of measures to improve the quality of maternity care during childbirth. Sufficient and well-organized physical environments in midwifery units are urgently needed to safeguard women’s privacy when discussing reproductive and other health concerns and support improvements in the provision of midwifery care. Ensuring adequate space to accommodate birth companions is particularly important since it has been shown to decrease the incidence of obstetric violence in low- and middle-income countries (ref is needed here). Policy developers and administrators need to prioritize training, monitoring, and peer support to curb disrespectful and abusive care during childbirth, which seems to be deeply rooted in the routine unorthodox treatment of childbirth. The study findings also suggest a need for improvements to college and university curricula to reinforce the knowledge and skills needed to deliver respectful, dignified health care. Lastly, further research is needed to develop and evaluate approaches to support changes in routine midwifery care where practices are contrary to standards of care to create the conditions for optimal maternal and infant outcomes.

## Conclusion

Despite WHO’s emphasis that women should have positive experiences during childbirth to encourage return to facilities for future births, negative experiences are still endured by women. They could lead to home births, consequently increasing avoidable maternal morbidity and mortality rates (WHO, 2018). The study findings showed that the current childbirth management in the study setting violates women’s rights to dignity, exposing them to life-threatening conditions that could have been avoided with improvements to midwifery education and practice. Innovative and respectful models based on best practices must be taught to replace and improve the current deleterious situation (Jiru & Sendo, 2021). In addition, improved physical layouts and resources in labor units are required to enable midwives to practice respectful maternity care.
